# Ex Vivo Human Histology Fractional Treatment with a New CO_2_ Scanner: A Potential Application on Deep Scarring

**DOI:** 10.3390/medicina59061117

**Published:** 2023-06-09

**Authors:** Paolo Bonan, Laura Pieri, Irene Fusco, Francesca Madeddu, Tiziano Zingoni, Claudio Conforti, Domenico Piccolo

**Affiliations:** 1 Laser Cutaneous Cosmetic and Plastic Surgery Unit, Villa Donatello Clinic, 50019 Florence, Italy; dr.pbonan@gmail.com; 2 El.En. Group, 50041 Calenzano, Italy; l.pieri@deka.it (L.P.); f.madeddu@elen.it (F.M.); t.zingoni@elen.it (T.Z.); 3 Dermatology Clinic, University of Trieste, Piazza dell’Ospitale 1, 34125 Trieste, Italy; claudioconforti@yahoo.com; 4 Skin Centers, 67051 Avezzano, Italy; f.domenico.piccolo.skincenters@gmail.com

**Keywords:** human histology, CO_2_ scanner, scar remodelling

## Abstract

*Background and Objectives*: For many years, fully ablative laser treatments, particularly those performed with a carbon dioxide (CO_2_) laser, were regarded as the gold standard for resurfacing. This study’s goal is to assess the depth that can be reached by a new CO_2_ scanner system, through a skin model with greater dermal thickness, to use in the treatment of deep scarring. *Materials and Methods*: Male human skin tissue was laser-treated using a CO_2_ fractional laser and a new scanning system, and all samples were fixed in 10% neutral buffered formalin, dehydrated using a series of crescent alcohol, embedded in paraffin, sectioned in series (4–5 µm thick), stained with haematoxylin and eosin (H&E), and then analysed under an optical microscope. *Results*: From the epidermis through the underlying papillary and reticular dermis to various depths of the dermis, microablation columns of damage and coagulated microcolumns of collagen were observed. The reticular dermis was fully penetrated up to 6 mm at higher energy levels (210 mJ/DOT), resulting in deeper tissue injury. Although the laser might penetrate further, the skin stops there, leaving just the fat and muscular tissue. *Conclusions*: The deep layers of the dermis can be penetrated by the CO_2_ laser system throughout the entire dermal thickness when using the new scanning system, suggesting that this laser’s potential impact, at the selected settings, covers all skin targets required to perform superficial or deep treatments on any dermatological issue. Finally, patients who have problems, such as morbid scar-deep complications, which affect their quality of life, are more likely to profit from this innovative technique.

## 1. Introduction

For many years, fully ablative laser treatments, particularly those performed with a carbon dioxide (CO_2_) laser, were regarded as the gold standard for resurfacing. Laser CO_2_ treatments are required because of significant skin remodelling and in the management of different scarring conditions [1,2]. Nonetheless, these invasive treatments can be associated with negative post-treatment outcomes such as pigmentary changes, swelling, erythema, blistering, crusting, and, in some cases, scarring, if not properly managed [3]. Fractional ablative lasers have a higher safety profile than traditional ablative lasers, with a reduction in erythema onset and recovery times [4]. These advantages, combined with the associated decrease in scarring and pigmentation issues, have played a role in the growing acceptance of fractional laser treatments. Fractional CO_2_ therapy results in a sort of microablation of the epidermis (MAZ, microscopic ablative zone) that heals in 24–48 h; and in a sort of microthermal zone (MTZ) found around the ablation zones. Keratinocyte migration and damaged cell extrusion at the borders of the ablative and heated zones speed up epidermal tissue regeneration [5]. With much less downtime and adverse effects as compared to conventional lasers, fractional ablative CO_2_ lasers can successfully promote skin regeneration. However, occasionally even fractional laser therapy might result in hyperpigmentation (PIH), correlated with the amount of energy employed [6]. Several studies have shown that fractional ablative CO_2_ lasers are an effective treatment for enhancing the function and appearance of burn scars [7,8,9], with improvements in the scars’ texture and colour [10]. 

Greater ablations and higher energy are both needed for deep scarring. To effectively treat the most severe scars, laser action depths of more than 1 mm must be used, which may be achieved by increasing the ablative power, correlated with the system’s energy output [11,12]. Inflammation risk, therefore, rises, since an increase in the depth of action is directly correlated with the amount of energy provided. 

The optical mechanism of the traditional scanner was enhanced by lowering the spot sizes, resulting in the creation of a new scanner (SCAR3, DEKA M.E.L.A., Florence, Italy). This reduced patient pain and the risk of the onset of PIH while allowing for deeper ablation depths and less energy delivery [13]. The CO_2_ family lasers (DEKA M.E.L.A., Florence, Italy) may be utilised with the SCAR3 scanner to emit an energy/DOT (DOT is the area that interests both MAZs and MTZs in the tissue) of over 600 mJ. The SCAR3’s CO_2_ laser emission is fractionated, resulting in DOT columns that are incredibly thin, widely dispersed, and penetrate very deeply into the epidermal layer. Ex vivo skin models have proved to be highly effective histologically, demonstrating the depths reachable by these devices [13]. However, for depths greater than 4 mm, it is important to have a model with enough skin thickness to not affect the measurements.

Given the scanner’s efficacy in both preclinical studies made with ex vivo animal models (sheep skin) and clinical studies on acne scars [13], this study’s goal is to assess the depth that can be reached by the SCAR3 scanner through a skin model with greater dermal thickness and to use it in the treatment of deep scarring.

## 2. Materials and Methods

### Ex Vivo Preclinical Test

To assess the action depth of the SCAR3 scanner, a histological evaluation was carried out. Due to its deeper thickness than sheep skin [13], human skin taken during abdominoplasty surgeries was employed. In particular, the male human abdomen was selected because it has the thickest skin layer [14].

We investigated the effect of the SCAR3 scanner on human histological samples using higher energies (140–210 mJ/DOT) than those presented in sheep preclinical data due to the limitation of the previous skin model [13]. 

Following laser exposure, all samples were fixed in 10% neutral buffered formalin, dehydrated using a series of crescent alcohol, embedded in paraffin, sectioned in series (4–5 µm thick), stained with haematoxylin and eosin (H&E), and then analysed under an optical microscope.

## 3. Results

### Preclinical Results with Histological Analysis

**Figure 1:**
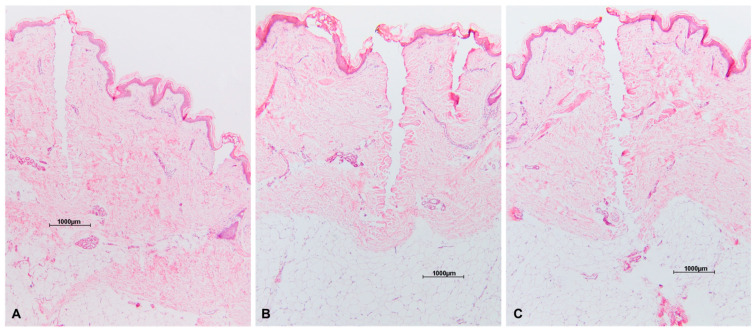
Energies/DOT 140 mJ (**A**), 150 mJ (**B**), 210 mJ (**C**). Changes in ablative, thermal, and total micron depths in ex vivo human skin after the SCAR3 treatment. Maximum Ablation Depth: 3909 µm (**A**), 4136 µm (**B**), and 5727 µm (**C**). Maximum Thermal Lesion Depth: 4318 µm (**A**), 4818 µm (**B**), and 5954 µm (**C**). (**A**–**C** scale bars 2×).

**Figure 2:**
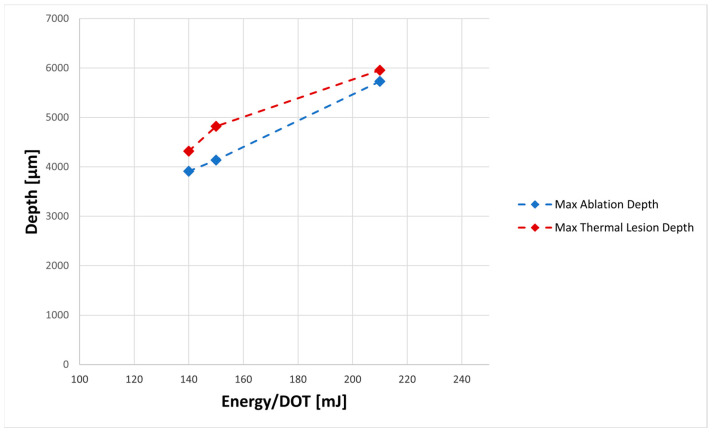
A plot of the depth as the energy increased: the curve with a red line is the Maximum Thermal Lesion Depth achieved with the SCAR3 scanner; the curve with a blue line is the Maximum Ablation Depth achieved with the SCAR3 scanner.

Human skin tissue was laser-treated using a CO_2_ fractional laser and the SCAR3 scanning system and H&E-stained slices of the tissue revealed narrow columns with increasing depth when energy/DOT (mJ) increased. From the epidermis through the underlying papillary and reticular dermis to various depths of the dermis, microablation columns of damage and coagulated microcolumns of collagen were observed (Figure 1 and Figure 2). The reticular dermis was fully penetrated up to 6 mm at higher energy levels (210 mJ/DOT), resulting in deeper tissue injury. Although the laser might penetrate deep further, the skin stops there, leaving just the fat and muscular tissue. The histological results on human samples with higher energies (140–210 mJ/DOT) continue the trend in ovine histology findings about the SCAR3 that were reported by Scarcella et al. in 2022 [13] (with energy/DOT 10–150 mJ due to the animal model limit in thickness). 

## 4. Discussion

In contrast to the usual omnidirectional type III collagen, scars are caused by a disorganized healing response that leaves behind fibrotic type I collagen. Collagen makes the wound stronger and makes it easier for macrophages and endothelial cells to move around it. When compared to healthy skin tissue, the ratio of collagen type I to type III is higher in mature scars. Older scars have fewer blood vessels and typically lack skin appendages such as sebaceous glands, sweat, and hair follicles. After an acute injury (such as surgery, a laceration, an abrasion, a burn, or a deep inflammatory skin disease such as acne), thickened raised scars develop due to excessive collagen synthesis (fibrosis). Through a real microablation, a CO_2_ laser with a fractional scanning system (SCAR3 scanner) generates micro columns of thermal damage surrounded by healthy tissue that can cause the vertical scaling of the epidermis; these microthermal zones (MTZs) found around the ablation zones encourage a new and rearranged collagen pattern that is crucial to the neocollagenesis process [15]. The skin around each column does not suffer thermal damage (ablation and coagulation), but only a biostimulation effect and evaluation of the distance between each single DOT is fundamental to achieving the fastest healing process and the best clinical result. Collagen stimulation and the cutaneous rejuvenation effect depend on the depth of thermal damage at the level of the dermis that triggers the formation of new collagen and tissue repair [16].

Additionally, if this is coupled with the SCAR3 scanning system’s ability to achieve a deeper level of action (owing to its tiny spot size) and a more noticeable bleeding and crusting process than a traditional scanner, an exceptional outcome is undoubtedly reached [13]. An increase in the coagulation area caused a greater shrinkage of the tissue but still fell within a range that did not affect the tissue healing. This innovative laser system enhanced its effects, both in terms of tone thanks to a greater tensor effect, and of stimulation because of a greater volumetric thermal effect. This greater shrinkage effect on the ex vivo skin model was already demonstrated in the study of Nisticò et al. [17].

The new scanner having smaller spot sizes may be optimal for rapid epidermal wound healing by minimizing the keratinocyte migration path or period. The fractional approach combined with an optimal treatment coverage or spot density and pulse energy may allow for a favourable ratio of viability to the treated tissue. 

Deep scars require more ablation and therefore also more energy. The most severe scars require lasers with a depth of action >1 mm, and this can be achieved by increasing the ablative power, which is related to the energy delivered from the system [18]. Since an increase in the depth of the action is directly related to the energy supplied, this consequently increases the risk of inflammation. This is why it was necessary to reduce the spot size of the DOT (columns of thermal harm). From this perspective, the new scanner SCAR3 was developed to obtain greater depths of ablation and, at the same time, reduce the amount of energy delivered to limit patient pain and the risk of occurrence of PIH.

The CO_2_ laser system, with the use of the new SCAR3 scanning system and conservative parameters, reaches the deep layers of the dermis up to a depth of >1 mm. This implies that the potential effect of this laser, at the chosen parameters, covers all skin targets necessary to carry out superficial or deep treatments for any dermatological problem. Indeed, many scar areas are more than 1 mm thick and extend more than several millimetres in depth. To effectively treat these lesions, a laser ablation penetration depth greater than 1 mm is required, going as deep as possible to initiate the healing cascade and achieve good results in scar remodelling [13].

In addition to guiding patient selection, the type of injury and the variation in penetration depths caused by ablative and thermal injuries of our study device also determine the type of skin scars to be treated, how many passes are made during a single treatment period, and whether additional treatments are necessary. The deep layers of the dermis can be penetrated by the CO_2_ laser system throughout the entire dermal thickness when using the new SCAR3 scanning system; this suggests that this laser’s potential impact, at the selected settings, covers all skin targets required to perform superficial or deep treatments on any dermatological issue. Many scar areas extend more than several millimetres deep, and so to start the healing cascade and achieve effective results in scar remodelling, a laser ablation penetration depth is necessary [13].

More significantly, this novel scanning technology enhances treatment effectiveness while also decreasing patient downtime and associated recuperation periods [13].

Our histological findings confirm that the SCAR3 scanner enabled us to treat a wide variety of skin lesions because it can penetrate skin lesions up to a depth of 5954 m, as is evident in Figure 1.

Patients who have problems such as deep morbid scar complications, affecting their quality of life, could benefit from this innovative technique. Our system may be applied clinically in many pathologies as it is possible to adjust the depth of action based on the energy delivered up to reaching depth values capable of treating different skin scars such as deep and conspicuous skin lesions/conditions. Therefore, expanding the plan and the findings of the study is our long-term objective.

## 5. Conclusions

The study’s findings confirm the Maximum Thermal/Ablation Lesion Depths achieved with the SCAR3 scanner and its dependence on energy delivered by the laser system. These findings will enable us to expand the clinical applications of the research tool to a variety of skin lesions.

## Data Availability

Data that support the study findings are available on request from the corresponding author (I.F).
